# Reply to “Factors Associated with Overutilization of Computed Tomography Cervical Spine Imaging”

**DOI:** 10.5811/westjem.18614

**Published:** 2024-02-28

**Authors:** Karl Chamberlin

**Affiliations:** * University of Massachusetts Chan Medical School, Emergency Medicine, Worcester, Massachusetts; † UMass Memorial Health, Department of Emergency Medicine, Worcester, Massachusetts

November 29, 2023

Dear Editor:

We appreciate the feedback and commentary on our recently published study. First, we would like to acknowledge the limitations of having a single reviewer performing all chart reviews. To mitigate potential bias, we created objective binary definitions for each NEXUS criterion. For example, we explicitly defined distracting injuries to include only radiographic findings of long bone fractures or multiple rib fractures, or any injuries that were described explicitly as “distracting” in the clinical documentation. Similarly, all documented neurological deficits were assumed to fulfill this criterion unless the clinical documentation explicitly ascribed the deficit to pain. While these definitions are more explicit and objective than the original NEXUS criteria, we felt this modification was necessary due to the retrospective nature of our study, as our methodology inherently prohibited us from prospectively asking clinicians whether an injury was distracting, for example. Furthermore, we felt that our definitions were aligned as closely as possible to the original criteria and, therefore, provided an accurate estimation of overutilization. Ideally, future studies on the topic should use multiple reviewers and prospectively collect data on the subjective criteria.

Secondly, we agree that a more granular analysis of mechanisms of injury could reveal additional associations with overutilization. In our analysis, none of the mechanisms of injury were significantly associated with CT overutilization, and seasonal mechanisms accounted for a very small percentage. Our categorization of mechanism was intentionally broad, as we sought to identify potentially meaningful targets for future interventions aimed at reducing overutilization. While it is possible that a subset of mechanisms within a given category could be statistically significant, this would require further investigation, and it is not clear whether those findings would yield clinically important targets for intervention.

Lastly, and most importantly, we would like to emphasize the letter writer’s point about flow and triage processes that may affect overutilization of CT resources. Because none of the sites included in this study use a clinician-in-triage process, we were unable to directly assess this association from our dataset. However, strategies to improve emergency department (ED) throughput (eg, clinician-in-triage staffing models, direct-to-CT protocols, nurse-initiated orders) have proliferated across the country in recent years, despite these strategies having uncertain impacts on resource overutilization. As we develop and implement novel flow and triage processes, it is paramount that we consider the secondary effects on healthcare costs, radiation exposure, incidental findings, and hospital resources. At a time when ED crowding and boarding have reached crisis levels, effective resource utilization is essential for operational success.

Thank you.

Karl Chamberlin, MD, MBA

